# Supplementary material for: Metamorphic pressure variation in a coherent Alpine nappe challenges lithostatic pressure paradigm
Source: Nat Commun. 2019 Oct 18;10:4734. doi: 10.1038/s41467-019-12727-z (PMC6800426; doi:10.1038/s41467-019-12727-z)
Supplement: Supplementary file 2 — Supplementary Information [file 41467_2019_12727_MOESM2_ESM.pdf]

Supplementary Information

Metamorphic pressure variation in a coherent Alpine nappe challenges lithostatic pressure paradigm

Luisier et al.

Supplementary Figure 1 Field observation of the transition zone between metagranite and whiteschist.

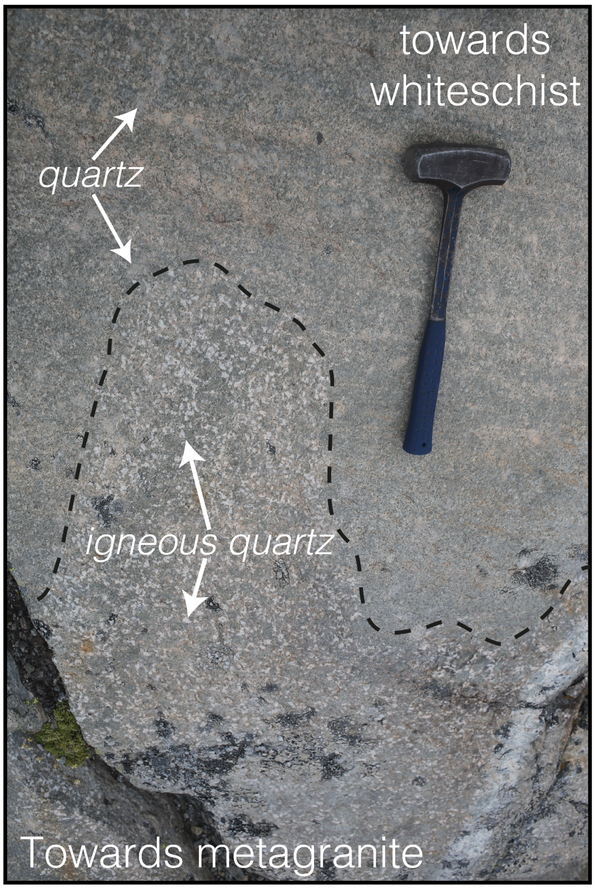

Field picture showing the irregular geometry of the transition zone between metagranite and whiteschist. The texture of the granite can be followed into the whiteschist. This is shown by milky-white igneous quartz grains in the transition zone, and some remnants of quartz in the whiteschist. The lobate, curved boundary is a typical texture for hydrothermally driven reaction fingering. This observation excludes the possibility, that the whiteschist is a xenolith. It confirms the hypothesis that the metagranite is the protolith of the whiteschist.

Supplementary Figure 2 Mass balance calculations of the whiteschist with a granite origin.

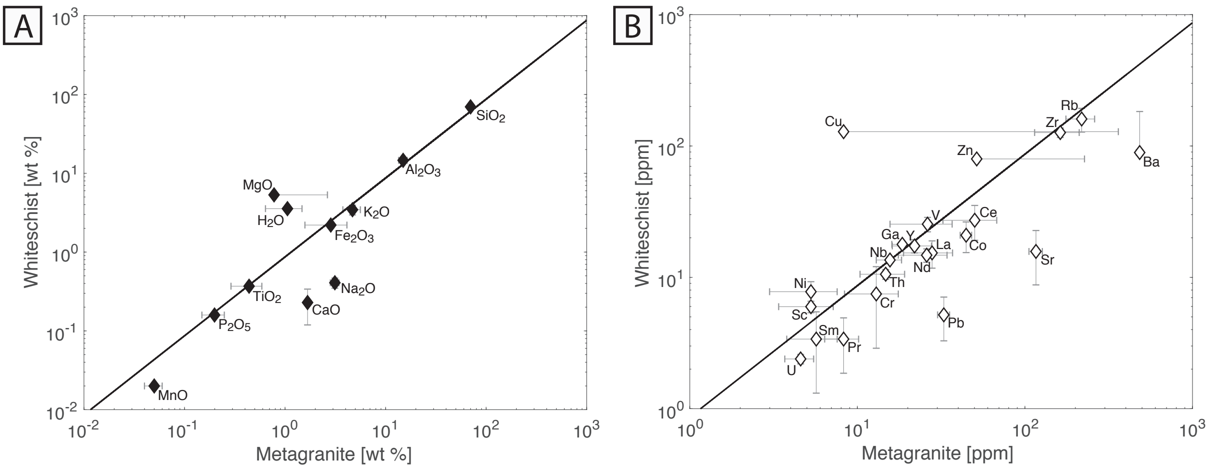

Results are presented in logarithmic ISOCON diagrams[1], showing the average composition of metagranite and whiteschist samples from the study of Pawlig and Baumgartner (2001, Table 1)[2], with A) major oxides in weight percent (wt %) and B) trace elements in ppm, of the same samples as in A). Note that data and error bars are displayed on logarithmic scales. Mobile oxides enriched in whiteschist are MgO and H_2O, whereas MnO, CaO and Na_2O are leached in whiteschist. All other major oxides, including SiO_2, are neither removed, nor added to the whiteschist chemistry. Hence, the metasomatic fluid was mainly oversaturated in MgO, undersaturated in CaO, Na_2O and MnO, and saturated in other major elements. Silica is an immobile element and was not added to the rock during metagranite alteration.

Supplementary Figure 3 Differential stress estimates from anorthite flow laws

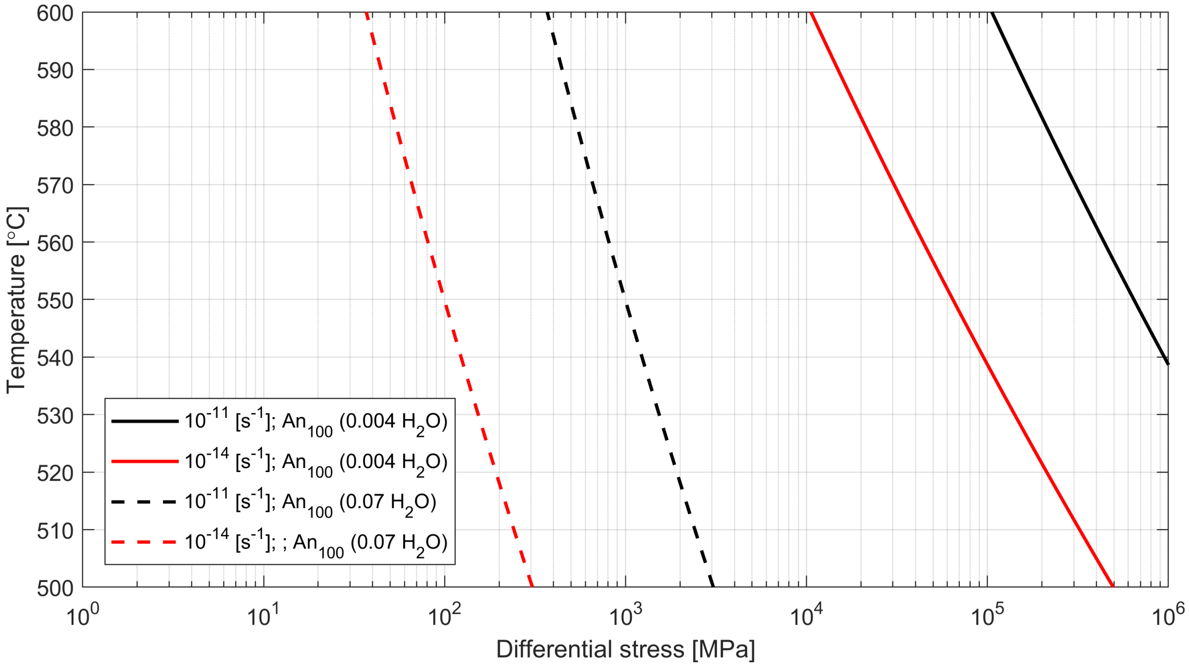

Differential stresses versus temperature for a range of strain rates and for two anorthites with different wt% of water. Flow laws and parameters from[3]. The experimentally derived flow laws[3] for dislocation creep for anorthite with wt% of H_2O of 0.004 and 0.07 have been used to plot the differential stress versus temperature for a range of strain rates. For a temperature range between 500 and 600 °C the differential stress calculation indicates that anorthite with wt% H_2O of 0.004, representing a "dry" feldspar specimen, would have differential stresses significantly larger than few GPas and would fracture. Anorthite with wt% H_2O of 0.07, representing a "wet" feldspar specimen, exhibits differential stresses around 1 GPa for the considered temperature range. If feldspar would have controlled the strength of the metagranite during a transient deformation event associated with nappe initiation, then differential stresses could have been ~1.4 GPa as used in the analytical solution for the pressure field. For "wet" feldspar the strain rates for stresses in the order of 1 GPa should be faster than ca. 10^{-12} s^{-1}. Therefore, the transient deformation phase of nappe initiation with high stresses should be short (<< 1 Ma) so that the metagranite remains little deformed. For example, for a strain rate of 10^{-12} s^{-1} the strain after thousand years of deformation is only ca. 3% (strain rate times duration).

Moreover, the transition from semibrittle to viscous creep deformation presumably occurs when the differential stress is equal to the effective confining pressure of the rock (so-called Goetze's criterion[4]). Therefore, assuming a differential stress of 1.4 GPa in a rock with a confining pressure of 1.4 GPa is feasible and would correspond to a stress state which is close to the onset of semibrittle deformation. The bulk strength of a natural feldspar-rich granite during tectonic deformation is simply unknown and laboratory derived flow laws for rock forming minerals can only provide estimates of the natural strength and stress levels. Based on Goetze's criterion and the presented flow laws for anorthite differential stresses in the order of 1 GPa seem at least possible locally and during a deformation phase of few thousand years.

Supplementary References

1. Baumgartner, L. P. & Olsen, S. N. A least-squares approach to mass transport calculations using the isocon method. *Econ. Geol.* **90**, 1261–1270 (1995).
2. Pawlig, S. & Baumgartner, L. P. Geochemistry of a talc-kyanite-chloritoid shear zone within the Monte Rosa granite, Val d'Ayas, Italy. *Schweiz. Mineral. Petrogr. Mitteilungen* **81**, 329–346 (2001).
3. Rybacki, E. & Dresen, G. Deformation mechanism maps for feldspar rocks. *Tectonophysics* **382**, 173–187 (2004).
4. Kohlstedt, D. L., Evans, B. & Mackwell, S. J. Strength of the lithosphere: Constraints imposed by laboratory experiments. *J. Geophys. Res. Solid Earth* **100**, 17587–17602 (1995).
